# Everybody's Page

**Published:** 1890-03-15

**Authors:** 


### Everybody's Page.

A Norwegian engineer has invented a machine which can pack one thousand boxes of matches in a minute.

Dr. Culbertson, of Zanesville, U.S., writing to the *Medical Record*, states that as long ago as 1861 he published the results of experiments on dogs and pigs, proving that in death from chloroform the first effect is upon the respiration, the heart ceasing to beat later.

Evil may be exaggerated very much in report, but there seems some truth in the statement that the women of our country indulge far too freely in narcotics and stimulants. Is not this fact only another reason for everybody to have some real object to attain in life? Really busy people have no time for miserable thoughts or self-indulgence, and so need no stimulant, for every moment in their day is filled up.

The miners' death-rate seems a much vexed question. Those who want to see the eight hours' Bill passed for underground workers say miners are a short-lived class. Dr. Speck tells us that the inhaling of pure coal-dust is not as injurious as is supposed, and the death-rate of miners only about 0·8 per cent., whilst for the general population it is much more. Numerous other testimonies show that miners are, as a rule, healthy and long-lived. Be this as it may, length of life is not all we want. The pleasure of living is something worth, and to work for hours cut off from the pure light of day can scarcely, we think, add to that pleasure.

Linen manufacturers would not be altogether grateful to Mr. Howard Young if his remedy for colds, influenza, and other trials came into general use. Our practice of using linen handkerchiefs, says Mr. Young, is foolish and short-sighted, and we ought to follow the example set us by the discerning Chinese, and use paper ones instead! By the constant use of the same handkerchief hour after hour, we are in danger of re-absorbing the bacillus, and a paper handkerchief could be burnt directly after use. Excellent idea; but when we are out-of-doors what are we to do with them? Mr. Young is silent on this point. The *Journal d'Hygiene* asks if the State is to erect small crematoriums on the highway for the incineration of our *mouchoirs*. Perhaps somebody else will come forward and help us in this difficulty.

The great god doctor of the Buddhist Siamese lived in the time of Buddha, and all the plants and herbs spoke to him, and were good enough to tell him certain magic words for dispelling different complaints. So when a Siamese is ill, he commits no such extravagance as sending for his medical man, he only utters a few words! But supposing the magic refuses to act? his friends then come to the conclusion that "he is possessed of a devil," and place little trays of rice and other delicacies outside the house, to induce the devil to come out and have a good feed, which is rather a reflection on the devil's little weakness. He had some good prescriptions, too, this old god-doctor; the bone of a certain fish's tail, the jaw of a hog, and many others. The pounded teeth of a tiger, an elephant, and a crocodile gave the courage of the first, the longevity of the second, and the solemn tranquillity of the third; what more could heart desire? This would have beaten the elixir of life. But the wisdom of this physician stopped short at rheumatism; even his skill could find no cure for that terrible pain which is caused by the "swamp spirit" clinging on to a person, and driving his nails in. The Siamese try and get rid of him by taking a sharp axe and chopping within a hair's breadth of their skin, so as to chop the devil off, but he doesn't seem to mind a bit, at least the poor Siamese are martyrs to rheumatism.

The extraction of oil from wood is becoming a most important industry in Sweden.

If we want to keep good-looking, we must keep good-humoured, and most of us are anything but anxious to help on the ravages Time brings along with him. It is the minor miseries, vexations, disappointments, and jealousies that sour the temper; scarcely, if ever, the real big troubles of life.

Cardinal Richard, the Archbishop of Paris, says that cremation is chiefly advocated by freemasons and other enemies of religion for the purpose of secularizing funerals, and he has issued a pastoral to this effect. This is a somewhat narrow view to take of cremation, and why it is more secular to burn than to bury it would puzzle anyone to say.

One of the most popular ways of curing obesity is certainly the use of the Turkish bath; it is a luxury, and no trouble; whereas banting, more or less, and exercise require a little self-denial. But, alas! for those who strive thus to regain the slim appearance of their youth, it is a clearly established fact that while weight may be considerably reduced during the sitting, compensation always occurs afterwards.

Advertising is not, as many suppose, an outcome of modern necessity, but is a very ancient practice; and the British Museum possesses a collection of old Greek advertisements printed on leaden plates. The Egyptians were great advertisers. Papyrus leaves over 3,000 years old have been found at Thebes, describing runaway slaves, and offering a reward for their capture—poor wretches; and at Pompei ancient advertisements have been deciphered on the walls. So perhaps, after all, the inhabitants of old Athens and Rome and many other ancient cities had to deplore the desecration of their fine buildings and places as much as we do in London at the present day.

The *New York Times*, in an article upon old text books, mentions, amongst others, one called "Physicking," published in 1750. The author informs his readers that "Cough can be cured in the beginning with riding moderately on horseback, and only taking some ground ivy tea, sweetened with syrup of horehound." "Consumption is a distemper slow and sure. Bleed 2 or 3 oz. every three days; also apply strong poultices under the arms. For inward medicines let him chew sassafras root. His diet should be an abundance of turnips, raisins, and licquorice; his drink, strong beer." We feel that such generous treatment would arrest consumption in anybody.

The views expressed by Dr. Hudson in his recent address at the Microscopical Society on "Some Difficulties in the Study of Natural History," such as "the frequent changes in classification, the ponderous technical language employed," etc., ought to be applauded by lovers of natural history of all ages. The study of natural history can never flourish unless it is made interesting, and this cannot be unless the study is practical. Some of us remember classes at school, botany, for example, which might have been made such a delightful hobby, and without our obtaining merely a superficial idea of the subject, our small brains might surely have been spared some of the endless dry classifications. So with natural history. The want of some expensive book, says Dr. Hudson, should not deter anyone from studying some group of creatures. The study of the living animal itself will provoke questions not answered in any book—such as where life first developed into consciousness, and why it did so, thus raising the companion of our leisure hours to the rank of an intimate sharer of our graver thoughts.

